# Supplementary material for: Is skin disinfection before subcutaneous injection necessary? The reasoning of Certified Nurses in Infection Control in Japan
Source: PLoS One. 2021 Jan 8;16(1):e0245202. doi: 10.1371/journal.pone.0245202 (PMC7794031; doi:10.1371/journal.pone.0245202)
Supplement: Attachment — Submitted filename: Review PLOS ONE 20200721 0517 skin disinfection before subcutaneous injection.docx [file pone.0245202.s001.docx]

**PLOS ONE –review 0517**

**Is skin disinfection before subcutaneous injection necessary? The reasoning of certified nurses in infection control in Japan**

This is a very important and relevant issue. Easy to read !

**Abstract :** Keywords:

Please put the words in alphabetical order

**Methods and Materials / Analysis**

**There are major flaws in the method part that need to be processed and written in a clearer way for the reader, please develop methods section for a better dependability and confirmability. Its most important for this studies credibility.**

- Clarify the design , inductive design?
- More distinct written about the inclusion and exclusions criteria
- Convenience sampling ? pureposeful sampling?
- How did you do when you contacted the nurses for asking them to participate? E-mail? Letter ? Phone call? How many nurses did you ask?
- I want a flow chart showing how the participants were included in the study.
- In-depth interviews ? You write that semi-structured I. was used.
- References missing in text .
- Explaine more about the interview situation . Witch demographic variables did you include in the questionnaire?
- Did you have any follow up questions? (To clarify some answers) like “Can you explain more?”, “How do you mean”? were asked. Did you ask the nurses the demographic questions before you started…
- Who did the transcribed verbatim. The authors ? A secretary?
- Was the interview guide developed for this study?
- Make a nicer table –

Dataanalysis

I cant’t follow you step by step in the preparation, organizing and resulting phases in the content analysis process. Qualitative content analysis process/method not clear described, what design have used according to your references?

I want a table presenting examples of the analysis process ;themes, subthemes, and quotations from the interviews. You must describe the process how these seven themes arise (dependability) It must be transparent.

The material needs to be further processed - seven themes and no subcategories?

I prefer Demographic in the method section. In what kind of ward did the Participants work? How often did they administrate a subcutaneous injection ? seldom ? often ? education level?

Result

The results section and the tables must also be presented more clearly, sometimes too many and long quotes -see even over that part. develop the flow chart and present this in the resultsection.

Discussion

**The results of the discussion must be problematized and developed further**

Some suggestions….

**I** think you must discuss the importance of the nurses following guidelines or not and if you have any national guidelines to follow ?? in such cases how do you implement it? Suggestions for clinical implications?

I n the ethical section discussion, were the participates informed that they could withdraw from the study at any time ?

What is your bullet points? What is new? knowledge gap ?

I think your results indicate a very important situation – however you must discuss your old references, today a very high figure with multi drug resistance exists when we discuss hospital and hospital care. Coagulase-negative staphylococci (CoNS) are the most abundant micro-organisms colonizing human skin and mucous membranes all been shown to alter the flora, reduce the variety of strains and result in accumulation of multi-drug-resistant CoNS,

I think this can be discussed more, I’m not convinced that the same guidelines should prevail in hospitals as well as in homes and at homecaresttings. The conditions are completely different.. This is a new important point to discuss.

•	Discuss the nurse education in relation to guidelines?.

•	Discuss de-implementation - - it is very hard to change a behavior.. de-implementation is a process for identifying and removing practices based on tradition and habits which lack adequate scientific support. In the pursuit of evidence-based health care, de-implementation of old routines is just as important as the implementation of new evidence. (Upvall MJ, Bourgault AM. De-implementation: a concept analysis. Nurs Forum. 2018.) https://doi.org/10.1111/nuf.12256. was shown for the commonly used antibiotics.

Try to make the conclusion more spot on , I think it is to long.
