# Supplementary material for: Is skin disinfection before subcutaneous injection necessary? The reasoning of Certified Nurses in Infection Control in Japan
Source: PLoS One. 2021 Jan 8;16(1):e0245202. doi: 10.1371/journal.pone.0245202 (PMC7794031; doi:10.1371/journal.pone.0245202)
Supplement: Attachment — Submitted filename: Response to Reviewers0906.docx [file pone.0245202.s002.docx]

Sep 5, 2020

Dr. Wen-Jun Tu

Academic Editor

PLOS ONE

1160 Battery Street

Koshland Building East Suite 225

San Francisco、CA 94111 United States

Re: Manuscript ID: PONE-D-20-14697

Thank you very much for your e-mail and review of the manuscript (PONE-20-14697) that we sent on May 17, 2020. We would like to thank you and the two reviewers for providing constructive comments regarding the improvement of the original manuscript. All changes have been made in response to you and reviewers’ suggestions and itemized responses to the individual reviewer’s comments are also attached. In addition, the entire manuscript was revised to solve grammatical problems and improve readability through detailed English proofreading. These revisions are also colored in blue in the main document.

Reviewer #1:

Q1. The chosen theme is important, connected to the context, traditions and cultures in different countries. Hygienical care of the skin during subcutaneous injections seems to be a discussion theme that has no end. However, I would like to ask you how the theme of hand hygiene is raised when the nurses choose to protect the skin with or without disinfection? How do the nurses discuss the consequenses of skin hygiene during subcutaneous injections with the patient? These could be mentioned in the discussion part.

A1. We appreciate that helpful suggestion. As you advised, it is essential for both nurses and patients to maintain hand hygiene, whether disinfected or not prior to subcutaneous injection. In particular, after educating patients to ensure that they wash their hands for their own hand hygiene, nurses should assess the need for skin disinfection, explain the pros and cons of skin disinfection to patients, and make a decision after gaining their understanding. This point has been added to the revised draft. (P. 28-29 lines 418 – 428).

Q2. According to the method: The study is qualitative. It could be more clear if the comparative method that you used is also mentioned in the abstract. You also use content analysis in the study. Is this right method to this study (together with the comparative method)? The manuscript could be more clear if there is for example a table where you present the results of the comparative part.

A2. The phrase “comparative method” was not appropriate. I appreciate that you pointed this out. We have revised this term and discussed the inductive analysis; we also updated the references. We also modified abstract. In order to express clearly how the themes were extracted, we created a table 2 that includes the quotations from the participants, subcategories, and themes (P. 11 lines 164 – 175 and table 2).

Q３.The figure is missing a short explanation what it presents.

A３. A short explanation has been added to the figure’s legend.  (P. 26-27 lines 379-388).

Reviewer #2:

Q1. There are major flaws in the method part that need to be processed and written in a clearer way for the reader, please develop methods section for a better dependability and confirmability. Its most important for this studies credibility.
I cant’t follow you step by step in the preparation, organizing and resulting phases in the content analysis process. Qualitative content analysis process/method is not clear described, what design have used according to your references?
The material needs to be further processed - seven themes and no subcategories?
The results section and the tables must also be presented more clearly, sometimes too many and long quotes -see even over that part.

A1. Thank you for pointing that out. As you said, the description of how the data were analyzed was inadequate, so we have revised the text. We analyzed the data inductively. We have modified the description of the analysis method, using references to follow the "content analysis process.” In order to express clearly how the themes were extracted, we have created a table that includes the quotations from the participants, subcategories, and themes. We also removed quotations from the participants in the results section. The quotations are shown in shorthand in Table 2 (P. 11 lines 164 – 175 and tabe2).

Q2. The results of the discussion must be problematized and developed further.

A2. In order to further develop the results of the discussion, we added the discussion as follows:

(1)Skin hygiene and patient instruction including hand antisepsis (P. 28-29 lines 418 – 428).

(2)About de-implementation (P. 30-31 lines 450-462).

(3)Nurse education related to the guidelines (P. 31 lines 463 – 467).

(4)The necessity for new guidelines at home care settings (P. 29 lines 428 – 431).

Academic Editor

Q1. Keywords: Please put the words in alphabetical order．

A1. We revised manuscript accordingly.  (P. 4 line 41).

Q2. Clarify the design, inductive design?

A2. This study had an inductive design, so have I modified the text to reflect that.  (P. 6 line 86).

Q3. More distinct written about the inclusion and exclusions criteria.

A3. We have added the inclusion criteria. The selection criteria were as follows: (1) worked as a staff nurse with more than 10 years of experience (2) acquired the CNIC qualification. We did not describe the exclusion criteria because there was no exclusions criteria. (P. 7 lines 97 – 100).

Q4. Convenience sampling ? pureposeful sampling?

A4. The data collection method of this research was convenience sampling, so we added that information.  (P. 7 line 100).

Q5. How did you do when you contacted the nurses for asking them to participate? E-mail? Letter ? Phone call? How many nurses did you ask?

A5. To contact the study candidates, at first, we contacted the director of nursing at the hospital where the CNIC worked by telephone and postal mail. The directors of a hospital’s nursing department introduced their hospital's CNIC to us. And we visited with ten CNIC and explained the purpose of this study and asked them for cooperation in the research. (P. 7 lines 100-109).

Q6. I want a flow chart showing how the participants were included in the study.

A6.The participant selection process was poorly documented. Therefore, we have described the selection process in detail. With this description, we decided that the flowchart was unnecessary. But if you disagree, I will respect your opinion and add a flowchart.

We added the following text: “The first author contacted the director of nursing at the hospital in which CNIC worked. We asked ten director of a hospital nursing department with over 300 hospital beds to introduce us to the CNIC by telephone and letter. One of the directors of a hospital’s nursing department declined to cooperate because the significance of the study was not understood. Eight directors of a hospital’s nursing department agreed to participate and introduced their hospital's CNIC to the researchers one by one. One Nursing Manager introduced two CNIC from her hospital. There are usually only one or two CNIC in hospitals. We visited with ten CNIC and explained the purpose of this study to the m and sought their voluntary participation.” (P. 7 lines 100-109).

Q7. In-depth interviews ? You write that semi-structured I. was used.

A7. Yes you are right. I conducted semi-structured interviews, so I deleted the phrase “in-depth” and I have added “semi-structured.” (P. 9 line 134).

Q8. References missing in text .

A8. We provided the missing references.  (P. 11 lines 164-175).

Q9. Explaine more about the interview situation. Whitch demographic variables did you include in the questionnaire?

A9. We added the interview situation. All participants requested to be interviewed at the hospital where they worked, so the researchers conducted their interviews accordingly (P. 9-10 lines 145-147). We also added that demographic variables were collected to at the beginning of the interview (P. 10 lines 153-155).

Q10. Did you have any follow up questions? (To clarify some answers) like “Can you explain more?”, “How do you mean”? were asked. Did you ask the nurses the demographic questions before you started…

A10. Yes, we followed up questions were asked during the interview. Therefore, we added about the follow-up question to the new manuscript (P. 10 lines 155-157).

Q11. Who did the transcribed verbatim. The authors ? A secretary?

A11. It was done by the first author who was the interviewer of all the interviews. (P. 10 line 159).

Q12. Was the interview guide developed for this study?

A12. Yes, it was. So we added the following text: The interview guide was developed for this study (P.10 lines 148).

Q13. Make a nicer table –

A13. I remade Table 1.

Q14. I cant’t follow you step by step in the preparation, organizing and resulting phases in the content analysis process. Qualitative content analysis process/method not clear described, what design have used according to your references?

A14. We did not clearly describe process of content analysis in detail. The revised manuscript was described in detail with references as follow.

“This study used an inductive content analysis [18]. The researchers read each verbatim transcript several times to obtain an overall understanding of the content and gain a sense of the whole [20]. The meaning units in the interviews related to nurses’ reasoning process were identified and coded. The codes were sorted into subcategories based on similarities and differences [19]. Depending on the relationships between subcategories, researchers can combine or organize them into larger headings [19].After assessing across subcategories, overarching themes were derived. When discrepancies in coding occurred, the investigators discussed and resolved them by consensus. The process was repeated until the content of each interview was compared with the content of all other interviews.” (P. 11 lines 164 – 175).

Q15. I want a table presenting examples of the analysis process ;themes, subthemes, and quotations from the interviews. You must describe the process how these seven themes arise (dependability) It must be transparent. The material needs to be further processed - seven themes and no subcategories?

A15. In order to understand the process of analysis of how the seven themes were extracted, the themes, subcategories, and quotations from interviews are shown in Table 2.

Q16. I prefer Demographic in the method section. In what kind of ward did the Participants work? How often did they administrate a subcutaneous injection ? seldom ? often ? education level?

A16. We have moved the description of the demographic to the methods section. And,　ｗe added the following in the method section: “One of the ten was a ward head nurse and one was a staff nurse. The other eight were not assigned to a ward but were assigned to a department that worked across the hospital as infection control specialists. The CNIC had all worked in several wards as staff nurses for more than ten years. Therefore, although there were participants who did not intervene frequently among co-workers, all participants were in a position to instruct nurses on infection control measures including skin disinfection before injection.” (P. 8 lines 112– 121).

Q17. The results section and the tables must also be presented more clearly, sometimes too many and long quotes -see even over that part. develop the flow chart and present this in the resultsection.

A17. We created Table 2 in the Results section.  The Interview quotations in the results section have been omitted from the main text of the paper and described in a concise form in the Interview quotations section of the table.

Q18. I think you must discuss the importance of the nurses following guidelines or not and if you have any national guidelines to follow ?? in such cases how do you implement it? Suggestions for clinical implications?

A18. It’s a question of whether nurses follow the guidelines, we think they should follow the guidelines. However, this study found that nurses comply with the guidelines in hospitals, but not in home care environments. In other words, we thought it was necessary to discuss whether the guidelines in the hospital setting and the guidelines in the home care settings should be the same, or whether they need to be developed separately. This point has been added to the revised draft. (P. 31 lines 436 – 467; P. 29 lines 428 – 431).

Q19. In the ethical section discussion, were the participates informed that they could withdraw from the study at any time ?

A19. I did explain to the participants that they could decline to participate in this study at any time, and I clarified this in the ethical considerations section.  (P. 8 lines 124 – 125).

Q20. What is your bullet points? What is new? knowledge gap ?

A20. What I would like to highlight the most was the following sentence in the first paragraph of the discussion. “Our study reviewed that CNIC's regarding registering disinfection different between hospitals and home care settings.” I have revised the first paragraph so that the reader can understand it.  (P. 27 lines 398 – 400).

Q21. I think your results indicate a very important situation – however you must discuss your old references, today a very high figure with multi drug resistance exists when we discuss hospital and hospital care. Coagulase-negative staphylococci (CoNS) are the most abundant micro-organisms colonizing human skin and mucous membranes all been shown to alter the flora, reduce the variety of strains and result in accumulation of multi-drug-resistant CoNS, I think this can be discussed more, I’m not convinced that the same guidelines should prevail in hospitals as well as in homes and at homecaresttings. The conditions are completely different.. This is a new important point to discuss.

A21. Thank you for your very constructive advice. Based on the results of the seven themes of this study and the results of previous studies, we thought that a home version of the guidelines should be developed. It should take into account the patient's physical condition and the environment at the time of injection, in addition to the guidelines applied in hospitals. In a previous study, the participants were outpatients, not hospitalized patients. At present, it has been pointed out that it is unclear whether omitting skin disinfection before subcutaneous injection is applicable to all patients. I mentioned this point in the 6th paragraph of the discussion. (PP. 28-29 lines 418 – 431).

Q22. Discuss the nurse education in relation to guidelines?

A22. We believe that there is a need for education regarding the description of the guidelines for disinfection before subcutaneous injection. However, in addition to this, it is necessary to introduce evidence on the necessity of skin disinfection and to educate the patients on the merits and demerits of disinfection and the necessity of individual assessment. These aspects have been added to the manuscript. (P. 31 lines 463 – 467).

Q23. Discuss de-implementation - - it is very hard to change a behavior.. de-implementation is a process for identifying and removing practices based on tradition and habits which lack adequate scientific support. In the pursuit of evidence-based health care, de-implementation of old routines is just as important as the implementation of new evidence. (Upvall MJ, Bourgault AM. De-implementation: a concept analysis. Nurs Forum. 2018.) https://doi.org/10.1111/nuf.12256. was shown for the commonly used antibiotics.

A23. Thank you for your advice on de-implementation. I read the paper you recommended. Our results clearly demonstrate the barrier and resistance of de-implementation in skin disinfection prior to subcutaneous injection. We also thought it was important to educate nurses in order to advance the de-implementation process. These are added to the discussion section. (P. 30-31 lines 450 – 467 ).

Q24. Try to make the conclusion more spot on, I think it is too long.

A24. We shortened the conclusion.  (P. 32-33 lines 486 – 499).

We have also modified the Data Availability statement.

We believe that we have addressed comments of editor and reviewers and hope that the revised manuscript is now acceptable publication in PLOS ONE. Thank you for your generous consideration.

Sincerely yours,

Yuko Yoshida

Hokkaido University, Faculty of Health Sciences,

Department of Fundamental Nursing

Kita 12 Nishi5, Kita-ku, Sapporo, Hokkaido 060-0812, Japan

Phone and FAX: +81-(0)11-706-3718

Email address: yuko790402@hs.hokudai.ac.jp
