# Supplementary material for: Is skin disinfection before subcutaneous injection necessary? The reasoning of Certified Nurses in Infection Control in Japan
Source: PLoS One. 2021 Jan 8;16(1):e0245202. doi: 10.1371/journal.pone.0245202 (PMC7794031; doi:10.1371/journal.pone.0245202)
Supplement: Attachment — Submitted filename: Respons to Reviewers_SKINDISINFECTION.docx [file pone.0245202.s003.docx]

Oct 31, 2020

Dr. Wen-Jun Tu

Academic Editor

*PLOS ONE*

Re: Manuscript ID: PONE-D-20-14697R1

Dear Editor:

Thank you very much for your e-mail and review of the manuscript (PONE-20-14697R1) that we sent on Sep 5, 2020. We are very grateful for your constructive and insightful comments regarding the improvement of the revised manuscript. All changes have been made in response to your and the reviewers’ suggestions and itemized responses to the individual reviewer’s comments are also attached herewith. In addition, the entire manuscript was revised to resolve grammatical issues and improve readability through detailed proofreading. These revisions are also highlighted in red in the main document.

I look forward to working with you and the reviewers to move this manuscript closer to publication in PLOS ONE. Thank-you for your consideration. I look forward to hearing from you.

Sincerely,

Yuko Yoshida

Hokkaido University Faculty of Health Sciences

Address: Kita 12 Nishi 5, Kita-ku, Sapporo, Hokkaido 060-0812, Japan

Tel.:+81-011-706-3718

Fax: +81-011-706-3718

Email: [yuko790402@hs.hokudai.ac.jp](mailto:yuko790402@hs.hokudai.ac.jp)

Question1:

Abstract: Start the A. by going straigt, with 1-2 sentences, to the corn substance, not to previous litterature. Include: The study was done in autumn 2013. Please revise the A. The study does not "explain" you need to use "describe". Same words that you have in the text. A. needs much more stringency. "However" is not a good word in the abstact.

Response1:

We modified the sentence in the first line, deleted the section on previous literature, and added the following: “even though there is insufficient evidence on the need for disinfection.” We added that “interviews were conducted with 10 CNIC in 2013.” We also corrected and used the term “describe” instead of “explain,” Additionally, we reviewed the entire paper and avoided the use of the word “explain.” We have changed the expression from “however” to “on the contrary” in the abstract. We have made the above modifications to improve the stringency of abstract.

Question2:

Keywords: several missing: Japan, s.c injections (not only inj), content analysis. "Bake in Qualitative" into the A.

Response2:

We added “Japan,” “subcutaneous injections,” “content analysis” as keywords. We were not sure what the phrase “Bake in Qualitative” into the A. means. Thus, we have not been able to correct for this. We are sorry to trouble you; we request you to kindly explain what you meant further. We will correct this as soon as you let us know.

Question3:

Introduction: Use same words throughout the whole paper. Now you use respondent, informant and participant at the same time, also interviewer and researcher. This is confusing. Reduce the use of the word "participant" and "therefore" (in the whole paper).

What is the main research question in this study? This should be clearly written.

Response3:

We apologize for the confusion caused by the use of “respondent,” “informant,” and “participant” interchangeably; we have corrected them all to “participant.” In addition, the term “interviewer” has been changed to “researcher” to make it easier to follow. We reduced the usage of “participant” and “therefore” throughout the paper (participant:41→38, therefore:11→6). Special attention was paid to the use of unified terms and selection of conjunctions while proofreading.

We have modified our research question in the introduction as follows. “The research questions in this study were why Japanese nurses are performing skin disinfection before subcutaneous injections and what is the background to the practice. Based on this research question, the purpose of this study was to describe nurses’ reasoning for disinfecting or not disinfecting the skin before administering a subcutaneous injection, using a qualitative design.” (P. 4 lines 78-82).

Question4:

Methods: Datacollection (DC). What about having a separate part for the study design and putting together DC and participants as a overhead. Now this text is mixed. Generally now its more clear. I think that you do not need a flow chart according to the participants.

Response4:

Thank you for your advice. We outlined the “study design” in one section and then delineated the “participants and data collection” section together.

Question5:

Ethical considerations (E): This part should be moved before the results part. Now it confuses the reader in the method part. Keep it as a separate part for to make the paper clear. You need references to the E. since human beings participated. Use for example Helsinki Declaration and some local ethical committee descriptions from Japan. This is important.

Response5:

We removed the ethical considerations section before the Results part and have added it as a separate section. We added the following sentence: “This study followed the guidelines set out by the 1975 Helsinki Declaration (2008 version). The study was approved by the Hokkaido University Graduate School of Health Sciences Ethics Committee and the ethics committee of the study site (13-51).” (P. 9 lines 177-185).

Question6:

Line 103-104 p. 6 is unclear.

Response6:

We believe our intended meaning was not conveyed due to a spelling error. We have corrected it; kindly let us know if you seek further clarity.

(Before) We visited with ten CNIC and explained the purpose of this study to the m and sought their voluntary participation.

(After) The researchers visited the 10 CNIC, explained the purpose of the study to them, and sought their voluntary participation. (P. 5 lines 110-P.6 lines111).

Question7:

Line 107, p. 6: ...were willing to participate in the survey... Do you mean in the study or what?

Response7:

We apologize for the confusion; we have revised “survey” to “study.”

(Before) Ten CNIC were willing to participate in the survey.

(After) The 10 CNIC agreed to participate in the study. (P. 6 line 144).

Question8:

Line 135-137. p. 8. This is irrelevant info. Please remove curriculum vitae information from the manuscript.

Response8:

We have deleted the section in adherence with your comment.

Question9:

Results: Where did the results come from? Please shortly introduce this in the ingress. Did the previous studies influence the results? Or the interviews with participants? What about your method?

Response9:

We added the information you require in the beginning of the Results section.

“The researchers conducted semi-structured interviews with 10 CNIC and performed an inductive content analysis. Seven themes emerged around the rationale of why CNIC performed skin disinfection before administering subcutaneous injections: ‘common practice,’ ‘adherence to hospital norms,’ ‘ingrained custom,’ ‘no detrimental effect,’ ‘presumed merit of omitting disinfection,’ ‘avoiding persistent suspicion of infection,’ and ‘lowered adherence to norms in home care settings.’ The meaning of each theme is elaborated upon, using direct quotations from the participants in Table 2.” (P. 9 lines 188-194).

Question10:

The results part has in general stepped backwords and it feels that the you have lost the abstraction level. The text is now repeating the figure and the table. Please go back to your original manuscript and discuss the presentation of the results in your research group. This needs stringency and revision.

Response10:

We have tried to use subcategory-based descriptions for each theme’s description to provide a convincing explanation of how the theme was generated. As a result, we believe the contents of the table were repeated many times and the abstraction level was lost. We discussed this again in the research group and in the main text; we have modified the main text to describe the essential meaning of each theme, avoiding simple repetition of the table contents as much as possible. In addition, for the item “Reasons to perform skin disinfection before subcutaneous injection,” the number of repeats of legend in Fig. 1 increased. We have made revisions to minimize the duplication of descriptions between the two.

Question11:

Discussion: This part includes also many repeatings. Here you should raise the level of abstaction even more and discuss the paper in whole. Please discuss this part with your reseach group and revise the text.

Response11:

We deleted the repetition of the sentence. To increase the level of abstraction, we rethought the structure of the discussion section and rewrote it. The content of the structure is as follows: (1) summary of the results of this study; (2) comparison of the concept of de-implementation with the results of this study; (3) reference to the de-implementation of skin disinfection observed in some cases at home; (4) justification of the de-implementation of skin disinfection at home; and (5) development of guidelines to facilitate the de-implementation process of skin disinfection before subcutaneous injections.

Question12:

Line 365, p. 23. Is this necessary to mention? Many readers know this.

Response12:

We deleted the points you deem unnecessary.

Question13:

Line 382, p 23. NB, the English Language.

Response13:

We deleted the points you have mentioned.

Question14:

In general: I recommend you to use a professional English Language control.

Response 14:

We shared your comments with a publication support services company and asked for an experienced proofreader to check the language.
