# Supplementary material for: Is skin disinfection before subcutaneous injection necessary? The reasoning of Certified Nurses in Infection Control in Japan
Source: PLoS One. 2021 Jan 8;16(1):e0245202. doi: 10.1371/journal.pone.0245202 (PMC7794031; doi:10.1371/journal.pone.0245202)
Supplement: Attachment — Submitted filename: Respons to Reviewers_SKIN.docx [file pone.0245202.s004.docx]

Dec 13, 2020

Dr. Wen-Jun Tu

Academic Editor

*PLOS ONE*

Re: Manuscript ID: PONE-D-20-14697R2

Dear Editor:

Thank you for your e-mail and review of the manuscript (PONE-20-14697R2) that we sent on Oct 31, 2020. We are very grateful for your constructive and insightful comments regarding the improvement of the revised manuscript. All changes have been made in response to the reviewers’ suggestions and itemized responses to the reviewer’s comments are also attached herewith. In addition, the entire manuscript was revised to resolve grammatical issues and improve readability through detailed proofreading. Additions are marked in red, and deleted text is tracked via the ‘Track Changes’ function in Microsoft Word. The line numbers in this document are based on the main manuscript with the revision history and revisions shown in-line. These revisions are also highlighted in red in the main document.

I look forward to working with you and the reviewers to move this manuscript closer to publication in PLOS ONE. Thank you for your consideration. I look forward to hearing from you.

Sincerely,

Yuko Yoshida

Hokkaido University Faculty of Health Sciences

Address: Kita 12 Nishi 5, Kita-ku, Sapporo, Hokkaido 060-0812, Japan

Tel.:+81-011-706-3718

Fax: +81-011-706-3718

Email: [yuko790402@hs.hokudai.ac.jp](mailto:yuko790402@hs.hokudai.ac.jp)

Q1:There har been challenges to understand your track changes and two type of colouring the manuscript (red and blue) and what these mean and also the underlining in tables. There are a few grammatical problems and also words that need to be written correctly. If you could do the suggested changes and present the manus without track changes it would be easier to follow your thoughts.

A1: We would like to apologize for this confusion. We have improved the readability of the revised manuscript by unifying the color and using the track changes function in Word.

Q2:Abstract (A): The first sentence is a result. I suggest that you remove the words "continue" and "even though". Could "evidence-based" research be possible to use to highlight research?
Read carefully the A once more and see if you could higher the abstraction level some more.

A2: Thank you for your advice. I removed “continue” and “even enough” following your advice. To improve the abstract, we have modified the research background to highlight the significance of this research using the term “evidence-based.” The corrections and additions are in red.

Line 19-25

“Nurses continue to disinfect the skin before administering subcutaneous injections as a standard process in clinical settings; despite evidence that disinfection is not necessary. To implement evidence-based practice, it is critical to explore why this gap between “evidence” and “practice” exists. This study aimed to describe the reasons offered by Certified Nurses in Infection Control (CNIC) in Japan for performing skin disinfection before subcutaneous injection. Adopting an inductive qualitative design, interviews were conducted with 10 CNIC in 2013.”

In addition, the discussion section has been modified to describe the interpretation of the results using expressions that correspond to the expression of the research background.

Line 29-38

“The themes (c) and (g) were cited as the main reasons affecting decision-making. The CNIC administered injections following skin disinfection in hospitals in accordance with hospital norms. On the contrary, outside the hospital, they administered subcutaneous injections without skin disinfection. All themes except (b) and (g) reflect the barriers and resistance to omitting skin disinfection, while (g) shows that it is already partly implemented in home care settings. It is necessary to create a guideline for skin disinfection before subcutaneous injection that considers the quality of life of patients at home, their physical conditions, and the surrounding environment at the time of injection, in addition to the guidelines applicable in hospitals.”

Q3:Introduction (I): Research question(s) are a bit unclear. You name two questions and write about one in same sentence. How many questions do you have in this study? This is very important that you write it/them clearly. I miss also the study context you are using. This could be a part of the research question(s). Use rather "is" than "were" in line 85. p 5.

A3: Regarding the research question, we mentioned two in our previous manuscript: “Why Japanese nurses disinfect the skin before administering subsidiary injections and what is the background to this practice,” but deleted “what is the background to this practice,” and unified research question. The corrections and additions are in red.

Line 95-96

“Thus, we pose the following research question: why do Japanese nurses disinfect skin before administering subcutaneous injections?*”*

For the study context, we rewrote the following text before the text of the research question:

Line 91-95

“Although omitting skin disinfection before administering subcutaneous injections is safe, reduces the burden on patients, and likely reduces costs, it has still not become standard clinical practice. Exploring the reasons for this lack of adoption in clinical practice in Japan will help us address obstacles to the introduction of new evidence.”

Q4:Line 58. "Some studies..." You need a better start for the sentence. "Some" is coming also in next sentence.

A4: We have modified the sentence as per your suggestion, and now included a direct in-text citation as the beginning of the paragraph.

Lines 54-55

“Dunleavy et al. and Hope, Hickman, Parry, and Ncube [7,8] report that not using an alcohol swab is a risk factor for skin and soft tissue infections.”

Q5:Line 79. Please name the author.

A5: Following your advice, we have included the names of researchers.

Lines 74-76

“A study conducted in Greece　by Theofanidis [13] indicates that nurses disinfect the skin before insulin injections as a longstanding medical ritual, although there is insufficient evidence on the need for disinfection.”

Q6:Line 96 p.6. What is the difference between perception and experience? This could be explained in the indroduction (with references).

A6: Thank you for your advice. The difference between “experience” and “perception” is now described in the introduction section, with quotations. Additionally, we included in the introduction section the reason for targeting CNIC’s “experience” and “perception.” The corrections and additions are in red.

Lines 77-99

“Nurses who do not have extensive knowledge of infection control may assume that skin disinfection before administration of subcutaneous injections prevents infection. According to recent books published in Japan on nursing techniques and skills, disinfection is necessary, while only a few books have introduced studies verifying that it is unnecessary [15]. In contrast, Certified Nurses in Infection Control (CNIC) receive certification from the Japan Nursing Association for specializing in infection control and having advanced nursing skills. CNIC have more experience, skill, and knowledge-based perceptions than other nurses regarding skin disinfection before subcutaneous injection. In this study, the word “experience” is defined as “practical knowledge, skill, or practice derived from direct observation of or participation in events or in a particular activity” [16], while the word “perception” is defined as “The way in which something is regarded, understood, or interpreted” [17]. Describing CNIC’s experience and perception of skin disinfection before subcutaneous injection was considered suitable for this objective.

Although omitting skin disinfection before administering subcutaneous injections is safe, reduces the burden on patients, and likely reduces costs, it has still not become standard clinical practice. Exploring the reasons for this lack of adoption in clinical practice in Japan will help us address obstacles to the introduction of new evidence. Thus, we pose the following research question: why do Japanese nurses disinfect skin before administering subcutaneous injections? Based on this research question, the purpose of this study was to describe, using a qualitative design, CNIC’s reasoning for disinfecting or not disinfecting the skin before administering a subcutaneous injection.”

Q7:Lines 101-108 are a part of introduction.
Go straight to the participants under the heading on line 100.

A7: Thank you for your comments. Lines 101–108 has been moved to the introduction section.

Lines 111-113

“To meet conditions similar to the skills required for a subcutaneous injection, the participant selection criteria were as follows: (1) worked as a staff nurse with more than 10 years of experience and (2) acquired CNIC qualification.”

Q8:Line 188: What do you mean with “larger headings”?

A8: We apologize for this expression. We have revised this sentence as follows.

Line 168-170

“Depending on the relationships among subthemes, a larger number of subthemes can be organized, or combined, into a smaller number of themes [22].”

Q9:Line 190: Who were the "investigators"? Do you mean researchers of this study?

A9: We have revised “investigators” to “researchers of this study.”

Lines 171-172

“When discrepancies in coding occurred, the researchers of this study discussed and resolved them through consensus.”

Q10:Ethical Considerations (EC): This part is much better now.
Line 214: "After this..." sentence is unnecessary here. Write 1-2 short sentencences in the end of EC about publishing the results, since you are now planning to publish this study. What did you explain about publishing to the participants?

A10: Thank you for your comments. Following your advice, we deleted the sentence, and added more explanation on publishing the results to the participants.

Lines 192-194

“When briefing potential participants, the researchers explained the purpose and requirements of the study, participants’ right to withdraw at any time without consequences, and possibility of the authors publishing the results.”

Q11:Table 2. I advice you to think about to use the terms themes and subthemes or categories and sub-categories instead of mixing these with each other. It has been challenging to see the table through track changes.

A11: We apologize for the confusion. We attribute this issue to using Excel. The manuscript submitted the time before last one was made in Excel. Therefore, it was created by using Word from the previous manuscript. We have corrected the terminology and made all the tables more readable.

Q12:Results (R): Line 307 p. 17. Are you sure this is a right heading here? I suggest to form this and think about the sub-theme here once again. So many track changes here has given challenges to understand the results.

A12: Thank you for your comments. We have reconsidered the subtheme of “lowered adherence to norms in home care settings,” since it consists of subthemes “acknowledgment of those who omit disinfection” and “unnecessary skin disinfection is a burden for patients at home.” We believe “tacit approval for not disinfecting in home care settings” would be a suitable theme. This is because nurses in hospitals never think about complying with the norm; in situations where the norm in home care settings is lax, they start to consider other priorities, and it can be interpreted that they are hesitant and even partially acquiescing in omitting skin disinfection. This has been revised as follows.

“Tacit approval for not disinfecting in home care settings”

Q13:Discussion (D): Start your discussion with your study objective to lead the readers back to your corn substance. I dont understand why the example of urinary tract infection and catheterization is here, line 400 p.26.

A13: Thank you for your advice. We have revised the section as follows.

Lines 304-309

“The purpose of this study was to describe certified nurses’ reasoning for disinfecting or not disinfecting skin before administering a subcutaneous injection. A qualitative inductive content analysis generated seven themes. Although extant research of the past 50 years has consistently shown that swabbing the skin with alcohol before administering a subcutaneous injection is unnecessary [11, 24, 25], in clinical settings, nurses continue to disinfect skin, as shown in the theme of “ingrained custom.”

The reasons given for the example of urinary tract infection and catheterization are as follows. Intermittent urethral catheterization requires a strictly sterile technique in the hospital, but not at home. This issue is hotly debated. In case of skin disinfection before subcutaneous injection, sterile technique is only required in hospitals, but this process can be omitted at home. There is always a risk of infection if sterile techniques are not used; even so, sterile techniques are not required when intermittent urethral catheterization is performed at home. The example of urinary tract infection and catheterization was presented as a good example as a result. We have revised the description below to convey our intention.”

Lines 338-344

“Intermittent urethral catheterization is one example of a difference in medical technique required in the hospital and in home care settings. It is often self-administered by patients at home, and the use of antiseptic solutions during insertion has been a subject of much debate [32]. Although the risk of a urinary tract infection is always present at urethral catheterization, many recent studies support the use of a clean, rather than sterile technique when patients insert intermittent urethral catheterization in the home environment [33].”

Q14:Lines 405-414 are marked with blue colour. Is there a special reason for this?

A14: We apologize for the confusion. There was no difference in the message depending on the color. This mistake has been corrected.

Q15:In the D: you should mention clearly your future visions about your study substance, what to do next?

A15: Thank you for this input. We have revised the implications and future scope of the study as follows.

Lines 358-361

“Based on the results of this study and previous studies, evidence-based health care guidelines should be developed for skin disinfection before subcutaneous injections that consider the quality of life of patients at home, their physical conditions, and the surrounding environment at the time of injection.”

Q16:Limitations: you could see a bit more for this and also the conclusion.

A16: Thank you for your advice. The revised manuscript now includes the limitations, as shown below.

Lines 383-390

“The results of this study suggest that nurses have different reasoning for skin disinfection in home care settings than in hospitals. The participants in this study were experienced nurses who worked in hospitals. However, nurses who work in home care settings may have different reasoning on this issue. Further studies should consider the latter group to more deeply explore why nurses disinfect skin before administering subcutaneous injections. A quantitative survey will be required to clarify the actual status of skin disinfection before subcutaneous injections in home care settings when developing guidelines.”

We accordingly modified our conclusion as well:

Lines 388-395

“Our study described CNIC’s reasoning for disinfecting or not disinfecting skin before administering subcutaneous injections. Followed by an inductive content analysis, seven themes emerged: “common practice,” “presumed merit of omitting disinfection,” “adherence to hospital norms,” “avoiding persistent suspicion of infection,” “no detrimental effect,” “ingrained custom,” and, “tacit approval for not disinfecting in home care settings.” Participants in this study acknowledged practicing home care subcutaneous injections without prior skin disinfection. Within hospitals, however, compliance with hospital norms, rather than judgment about individual patient conditions, prevails. This study reveals the barriers and resistance to promoting evidence-based practice in skin disinfection before subcutaneous injections at clinical settings. Overall, hospital norms had the most influence on CNIC’s decision to disinfect.”

In addition, the entire manuscript was revised to resolve grammatical issues and improve readability through detailed English proofreading.
